# *Saussurea talungensis* (Asteraceae), a new species from Humla, Nepal Himalayas

**DOI:** 10.3897/phytokeys.176.61996

**Published:** 2021-04-16

**Authors:** Hum Kala Rana, Santosh Kumar Rana, Hang Sun, Kazumi Fujikawa, Dong Luo, Laxmi Raj Joshi, Suresh Kumar Ghimire

**Affiliations:** 1 Key Laboratory for Plant Diversity and Biogeography of East Asia, Kunming Institute of Botany, CAS, #132 Lanhei Road, Kunming 650201, China Kunming Institute of Botany Kunming China; 2 University of Chinese Academy of Sciences, Beijing 100049, China University of Chinese Academy of Sciences Beijing China; 3 The Kochi Prefectural Makino Botanical Garden, 4200-6 Godaisan, Kochi, Japan The Kochi Prefectural Makino Botanical Garden Kochi Japan; 4 National Trust for Nature Conservation, Bardia Conservation Programme, Bardia, Nepal National Trust for Nature Conservation Bardia Nepal; 5 Central Department of Botany, Tribhuvan University, Kirtipur, Kathmandu, Nepal Tribhuvan University Kathmandu Nepal

**Keywords:** Nepal, new species, phylogenomics, *
Saussurea
*, Talung valley, taxonomy

## Abstract

A new species *Saussurea talungensis* S.K.Ghimire & H.K.Rana, **sp. nov.** (sect. Strictae), from Talung valley of Humla district, Nepal, is described and illustrated. Morphologically, this species resembles *Saussurea roylei* and *Saussurea lanata* in habit, though it can be distinguished in having longer leaf petioles, purplish leaf margin, 1 or 3 capitula, shorter phyllaries, shorter receptacle bristles and the same anthers, comparatively shorter corolla with shorter lobes. Phylogenomic analysis also supports *S. talungensis* as a distinct species of *Saussurea*. Here, we provide taxonomic note, distribution map and phylogenomic inference to distinguish the new species and its allied members.

## Introduction

Genus *Saussurea* DC. is one of the largest and species-rich taxa in the Asteraceae (Shi and von Raab-Straube 2011). Geographically, it is confined to the high mountains of Central and East Asia, including the Himalaya but also occurs in arid highlands and open vegetation types elsewhere in the Northern Hemisphere (Wang et al. 2009). Chen (2015) reported ca. 458 species and von Raab-Straube (2017) approximated the species number to be 493. Among them, Nepal represents 44 species including 8 endemics (Rana et al. 2018; Tiwari et al. 2019). Recently, a number of new species were discovered from the Himalaya and Central/East Asia, including *S. ramchaudharyi* S.K.Ghimire & H.K.Rana, *S. bogedaensis* Y.J.Wang & J.Chen, *S. balangshanensis* Y.Z.Zhang & H.Sun, *S. yiwuensis* L.Q.Zhao & X.Ri, *S. yillingii* Y.S.Chen, *S. sagittifolia* L.S.Xu, S.Y.Yi & Y.S.Chen and *S. xinjangensis* Y.S.Chen & L.S.Xu. However, explorations are insufficient in many remote regions of Nepal compared to the neighboring region e.g., China.

**Figure 1.**
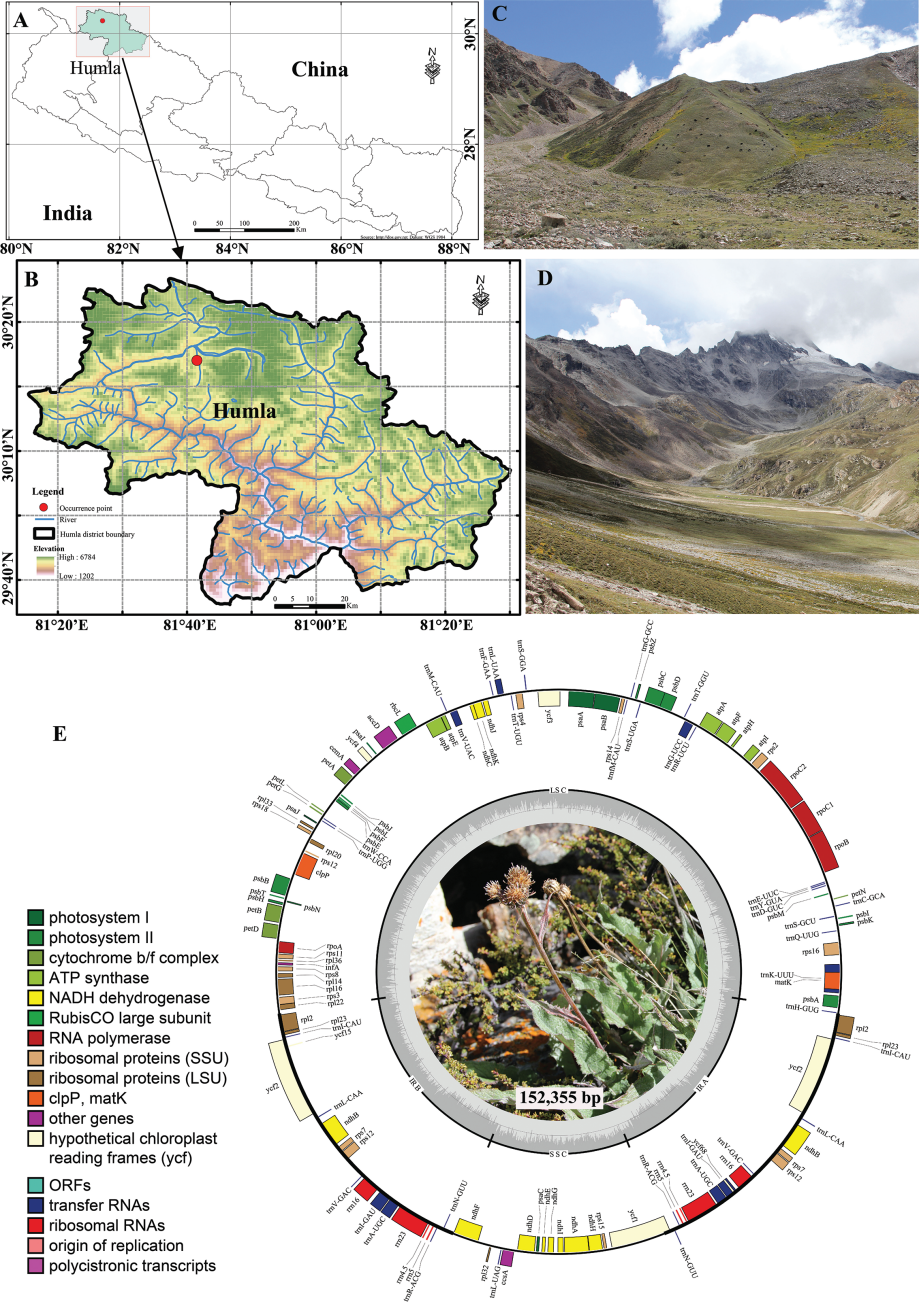
Distribution of *Saussurea talungensis* S.K.Ghimire & H.K.Rana **A, B** distribution map showing type locality in Nepal and Humla district respectively **C, D** type locality habitat between Nyalu Pass and Ning Tsho, Talung valley, Humla district **E** quadripartite plastome map of *S. talungensis* (Photographs **C, D** and plant picture in **E** by S.K. Ghimire).

A distinct population of *Saussurea* (Fig. 1) was recognized in 2012, during a botanical expedition to the alpine region of Humla district (NW Nepal). Based on habit, this population of *Saussurea* was initially considered as *S. roylei* (DC.) Sch.Bip. with some extent of resemblance with *Saussurea lanata* Y.L.Chen & S.Y.Liang. However, the population from Talung valley could not be ascribed to any known species of the genus *Saussurea* and may represent a new species. Therefore, in order to clarify this presumption, we characterized its morphology and clarify its genetic distinctness using chloroplast genome sequence. In particular, with the advent in the next-generation sequencing (NGS) technology, DNA-barcoding provides a rapid and precise solution for honing plant taxonomies when combined with more traditional, morphology-based approaches (Kress et al. 2005; Hajibabaei et al. 2007; Hollingsworth 2011; Ji et al. 2020). Integratively, morphological observations and molecular analysis led us to conclude that this population belongs to a new previously undescribed species of *Saussurea* (see below). We thus formally presented the description of the new species, *Saussurea talungensis* S.K.Ghimire & H.K.Rana.

## Methods

Three *Saussurea* specimen were collected in September 2012 from the type locality, Talung valley, Humla district in NW Nepal (Fig. 1A–D). The specimens were used for morphological and phylogenomic inference. A distribution map was produced using the type locality coordinates (Fig. 1A, B).

### Morphological observations

Morphological characteristics were described based on both observation and measurement collected with a ruler, calipers and electronic digital compound microscope. For the comparative morphological characteristics of allied taxa (*S. roylei* and *S. lanata*), related literatures (Chen et al. 1981; Shi and von Raab-Straube 2011; Chen 2014, 2015), live plant images and herbarium, and digitized specimen images from E, GH, K, KATH, PE, BM (acronyms following Thiers 2020, continuously updated) and CVH (www.cvh.ac.cn) were consulted.

### Plastome sequencing, assembly, annotation and phylogenomic analyses

Total genomic DNA was extracted from ~20 mg herbarium leaf tissue using a modified cetyltrimethylammonium bromide (CTAB) method (Doyle and Doyle 1987). A 500 bp DNA TrueSeq Illumina (Illumina Inc., San Diego, CA, USA) sequencing pair-end libraries were constructed using 3–5 μg sonicated DNA, according to the manufacturer’s instructions. The libraries were pair-end sequenced on the Illumina HiSeq 2000 platform. Raw reads were subsequently filtered to remove the low-quality reads and adaptors using the NGS QC Toolkit (Patel and Jain 2012), setting the cut off value for percentage read length to 80 and Phred quality score to 30. Remaining high-quality reads were assembled de novo to generate complete plastome with GetOrganelle pipeline developed by Jin et al. (2020). All the reads were then reference-assembled against the plastome of *S. hookeri* C.B.Clarke (MK952740) to check if the genomes were correctly assembled. The consensus sequence was annotated using *S. hookeri* as a reference in GENEIOUS v.7.0.2 (Kearse et al. 2012) and then corrected manually for the start/stop codons and intron/exon boundaries. Finally, the annotated plastid genome was submitted to GenBank (MW524864) and a physical map of the circular plastome was visualized with OrgannellarGenomeDRAW (OGDRAW: Lohse et al. 2013). To determine the phylogenetic position of the new species within the genus *Saussurea*, 64 plastome and a *rbcl* sequence (of *S. roylei*) of the genus *Saussurea*, plus one each for *Hemisteptia lyrata* (Bunge) Fisch. & C.A.Mey. and *Aucklandia costus* Falc. were accessed from GenBank (Table 1) and aligned with the newly generated sequence of *S. talungensis* in MAFT-WIN v.7.221 (Katoh and Standley 2013; Yamada et al. 2016). From the initial alignment, we selected conserved blocks with GBLOCK v.0.91b (Castresana 2000). We used GTR+I+G as the best fitting substitution model based on the Akaike information criterion (AIC) using JMODELTEST v.2.1.6 (Posada and Crandall 1998). We performed Bayesian phylogenetic inference (BI) analysis in MRBAYES v.3.2.6 (Ronquist and Huelsenbeck 2003) on online CIPRES Science Gateway v.3.3 (Miller et al. 2010; https://www.phylo.org). For BI, two independent analyses of four Markov Chain Monte Carlo (MCMC) chains were run for 5 × 10^7^ generations each with sampling every 1,000 generations. We assessed the stationarity of the runs using TRACER v.1.7 (Rambaut et al. 2018) and generated a majority rule consensus after removing a 20% burn-in. Maximum Likelihood (ML) analysis was performed using the graphical front-end RAXML GUI v.1.5b2 (Silvestro and Michalak 2012) in RAXML v.8.2.x (Stamatakis 2014) with 1,000 rapid bootstraps with 10,000 maximum number of trees. The Bayesian posterior probability (PP) from BI and Likelihood bootstrap support (BS) from ML of each branch was obtained. Nodes with PP ≥ 0.95 (Ronquist and Huelsenbeck 2003) and BS ≥ 75% (Hillis and Bull 1993) were considered well-supported.

**Table 1.** Accession numbers of the allied taxa of *Saussurea* and outgroups for the phylogenomic analysis (https://www.ncbi.nlm.nih.gov/).

Species name	Accession numbers	Species name	Accession numbers	Species name	Accession numbers	Species name	Accession numbers
*Aucklandia costus*	MH926063	*S. gossipiphora*	MH926100	*S. obvallata*	MH926128	*S. simpsoniana*	MH926162
*Hemisteptia lyrata*	MH926066	*S. henryi*	MH926103	*S. pachyneura*	MH926131	*S. sobarocephala*	MH926163
*Saussurea alaschanica*	MH926068	*S. hookeri*	MK952740	*S. pagriensis*	MH926132	*S. stella*	MH926166
*S. amara*	MH926070	*S. hylophila*	MH926104	*S. paleacea*	MH926133	*S. subtriangulata*	MH926169
*S. andryaloides*	MH926073	*S. involucrata*	MH926106	*S. peduncularis*	MH926135	*S. tangutica*	MH926173
*S. baicalensis*	MH926075	*S. japonica*	MH926107	*S. picridifolia*	MH926137	*S. thomsonii*	MH926174
*S. bhutanensis*	MH926078	*S. katochaete*	MH926110	*S. poochlamys*	MH926140	*S. tianshuiensis*	MH926176
*S. bracteata*	MH926080	*S. lanata*	MH926114	*S. przewalskii*	MK953475	*S. tomentosa*	MH926177
*S. centiloba*	MH926083	*S. langpoensis*	MH926115	*S. pseudoleucoma*	MK953469	*S. tridactyla*	MH926178
*S. chabyoungsanica*	MH926084	*S. laniceps*	MH926116	*S. pseudorockii*	MH926146	*S. tsoongii*	MH926179
*S. delavayi*	MH926090	*S. leontodontoides*	MK953477	*S. pseudosimpsoniana*	MH926147	*S. uliginosa*	MH926181
*S. durgae*	MK953478	*S. lhozhagensis*	MK953470	*S. pubifolia*	MK953467	*S. uniflora*	MH926182
*S. eriocephala*	MH926094	*S. licentiana*	MH926119	*S. roylei*	JQ933469	*S. velutina*	MH926184
*S. eriostemon*	MH926095	*S. malitiosa*	MH926122	*S. salwinensis*	MK953474	*S. wellbyi*	MH926185
*S. fuscipappa*	MH926096	*S. mucronulata*	MH926124	*S. semiamplexicaulis*	MH926158	*S. woodiana*	MH926186
*S. glabrescens*	MH926098	*S. nigrescens*	MH926126	*S. semifasciata*	MH926159	*S. xiaojinensis*	MH926187
*S. gnaphalodes*	MK953473	*S. nuda*	MH926127	*S. semilyrata*	MH926160		

## Results and discussions

### Taxonomic treatment

#### Saussurea talungensis

Taxon classificationPlantaeAsteralesAsteraceae

S.K.Ghimire & H.K.Rana sp. nov.

CF67BDBB-BB55-5226-A624-5BB6CF415A49

urn:lsid:ipni.org:names:77216567-1

Figs 1 , 2 , 3

##### Type.

Talung valley, between Nyalu Pass and Ning Tsho, open gravelly or stony slopes, 30.234°N, 81.692°E, 4300 m a.s.l., 13 September 2012, *S.K. Ghimire, A. Poudel, L.R. Joshi, S. Lo, P. Subedi, & C. Thapa CHH-1352* (***holotype***: KATH!; ***isotypes***: TUCH!, KUN!).

##### Description.

Perennial herb, caespitose, 22–50 cm tall. Caudex branched, stout, apex covered with petioles’ residues. Stem well-developed leafy, erect, simple, stiff, > 1.3 cm in diameter, purplish-brown at maturity, covered with brownish-white tomentose hairs. Basal leaves petiolate; petioles 9.0–10.5 cm; leaf blades lanceolate, chartaceous, 10–15 × 2.5–4.0 cm, adaxially green, with brownish-white tomentose hairs, abaxially greenish-white, with dense white tomentose hairs, base attenuate, margin purplish, sinuate-dentate to shallowly pinnately lobed, lobe margins entire, apex acute to acuminate, midvein distinct, purplish-green. Cauline leaves 5–7, gradually decreasing in size upwards, margin purplish; lower cauline leaves petiolate, petiole to 4 cm, leaf blades lanceolate, 8.0–11.5 × 1.5–2.5 cm, apex acute or acuminate; middle and upper cauline leaves subsessile to sessile, narrowly lanceolate to linear, 4.0–7.5 × 0.6–1.2 cm, undivided, margin dentate, purplish-green uppermost leaves subtending the capitula or synflorescence. Capitula 1 or 3 (2 not seen), shortly pedunculate to subsessile, tomentose. Involucres campanulate, 1.2–2.0 cm in diameter. Phyllaries in 4 to 5 series, imbricate, densely tomentose, apically purplish, acuminate, spreading to reflexed; outer phyllaries ovate-elliptic, 7–10 × 3.0–3.5 mm, middle phyllaries elliptic, 11–13 × 2.5–3.0 mm, inner phyllaries narrowly elliptic to linear, 13–15 × ca. 2 mm, only tips densely tomentose. Receptacles with bristles, ca. 4 mm long. Florets > 20; corolla purplish, 10.0–12.5 mm long; limb 4–6 mm including 1.5–2.2 mm lobes; tube 5–7 mm long. Anthers ca. 5 mm long, tails lanate, ca. 1.2 mm long. Style branches ca. 1.2 mm long, reflexed, short, papillate. Achenes cylindrical, 3.5–4.5 × 1.5–2.0 mm, ribbed, glabrous, apex shortly crowned. Pappus in two rows, pale brown; outer bristles 3.0–4.5 mm, scabrid, deciduous; inner bristles 10–12 mm long, plumose, persistent, sub-equaling floret.

##### Phenology.

Flowering and fruiting from July to September.

##### Etymology.

The specific epithet is derived with reference to the type locality of *Saussurea talungensis*, Talung valley, Humla district, NW Nepal.

##### Distribution and habitat.

*Saussurea talungensis* is currently recorded only from the type locality in Talung valley (between Nyalu Pass and Ning Tsho), Humla district, NW Nepal (Fig. 1A–D). It grows on the alpine open gravelly or stony slopes at an elevation ca. 4300 m a.s.l. (Fig. 1C, D).

##### Conservation status.

*Saussurea talungensis* is restricted to a single mountain and is represented by ~50 mature individuals within an area of < 500 m^2^ and appears to be local endemic. Moreover, its habitat and the population are subjected to high anthropogenic pressure, due to livestock grazing, and harvesting of caterpillar fungus and other aromatic plants used in medicine. Owing to population size, isolated distribution and observed constraints on the habitat due to anthropogenic pressure, *Saussurea talungensis* should be categorized as Critically Endangered [CR; B1ab (iii), B2ab (iii) and D] according to the IUCN Standards and Petitions Committee (2019).

##### Morphological affinities.

**Figure 2.**
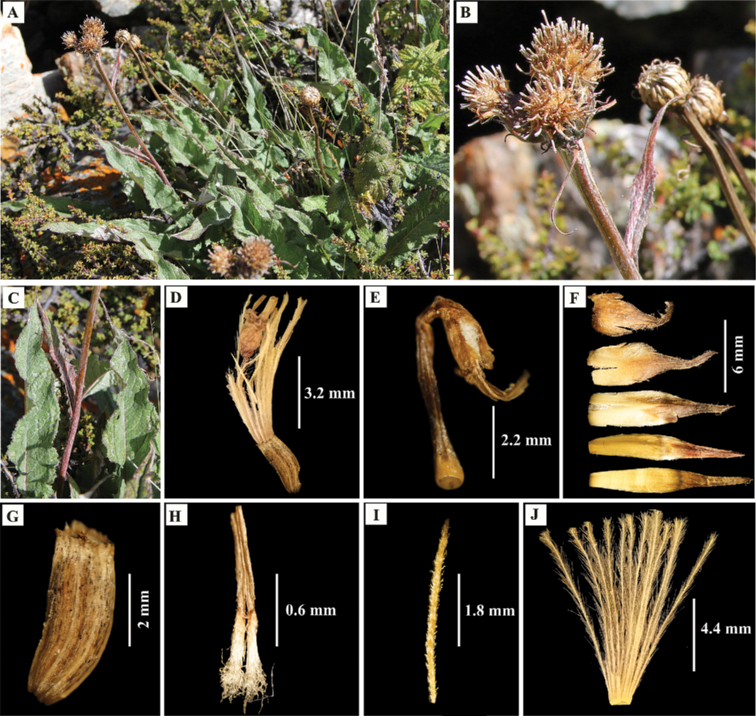
Live plants and microscopic photographs of *Saussurea talungensis* S.K.Ghimire & H.K.Rana **A** habit **B** synflorescence **C** leaf showing adaxial and abaxial surfaces **D** floret with pappus **E** exposed floret showing anthers **F** phyllaries (outer to inner, from top towards bottom) **G** achene **H** stamens **I** bristle of outer pappus **J** inner pappus. (**A–C** by S.K. Ghimire and **D–J** by H.K. Rana and S.K. Rana).

**Figure 3.**
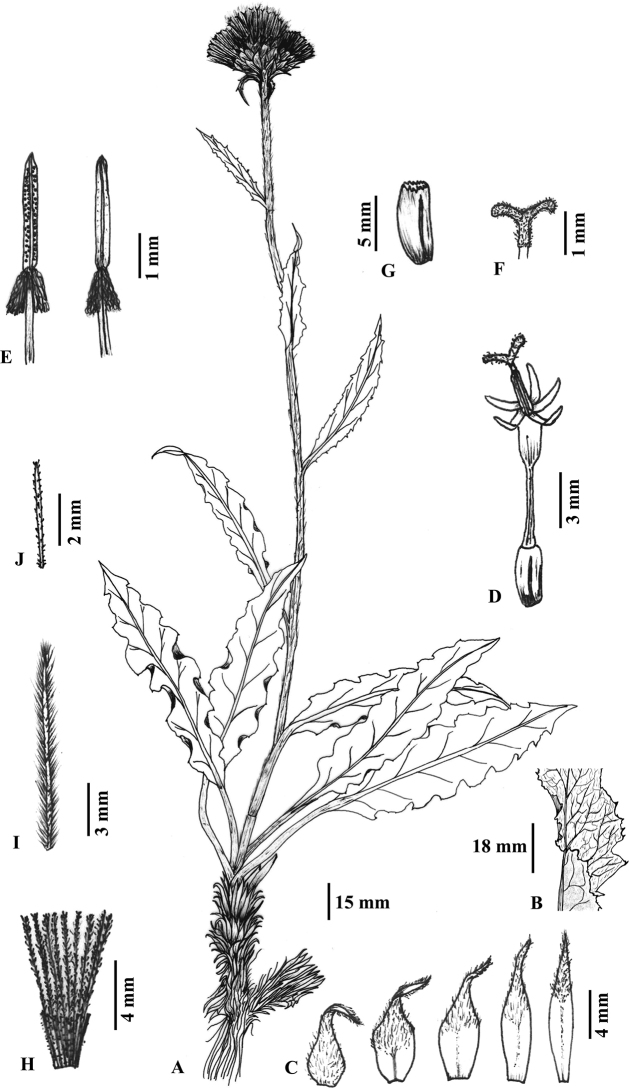
Illustration of *Saussurea talungensis* S.K.Ghimire & H.K.Rana based on the holotype **A** habit **B** leaf showing adaxial and abaxial surface details **C** phyllaries (outer to inner from left to right) **D** floret **E** stamens **F** style branches **G** achene **H** pappus **I** bristle of inner pappus **J** bristle of outer pappus (Drawn by H.K. Rana and S.K. Rana).

Critical examination of collected specimens, comparison with type material of allied taxa and relevant taxonomic literature revealed that *S. talungensis* is a new member of Saussurea (sect. Strictae). Based on morphology, distribution and ecology, this population of *Saussurea* was initially considered as *S. roylei* from sect. Strictae. To a certain extent it also resembles *S. lanata* in being a perennial herb with well-developed leafy stem, leaf blade undivided but lanceolate, many series phyllaries, campanulate involucres with more than 1 cm diameter, lanate anther tails, ribbed and glabrous achenes, and two rows of pale brown pappus. However, it differs from its allied taxa in having a number of qualitative and quantitative characters (see Table 2; Figs 2, 3). Furthermore, in the western Himalayan alpine region, *S. roylei* is considered to have diverse morphological variations but this proposed new species owned peculiar affinities which undoubtedly differentiates it from the stated and other *Saussurea* species.

**Table 2.** Character comparison of *S. talungensis* S.K.Ghimire & H.K.Rana and its allied taxa.

Characters	*Saussurea talungensis*	*Saussurea roylei*	*Saussurea lanata*
**Stem diameter / forms**	> 1.3 cm / stiff	≤ 1 cm / less stiff or herbaceous	≤ 1.2 cm or more / stiff
**Basal leaf size / petiole**	10–15 × 2.5–4.0 cm / 9.0–10.5 cm	7.5–25.0 × 0.5–2.0 cm / to 5 cm rarely up to 8 cm	7–28(–30) × 1.5–4.7 cm / 4–9 cm
**Leaf blade**	lanceolate, comparatively broader, chartaceous, margin purplish, base attenuate	lanceolate, chartaceous, margin green, base attenuate	oblong to narrowly elliptic, coriaceous, margin green, base decurrent
**Capitula number**	1 or 3 (2 not seen)	usually 1, rarely 2	1 to 3
**Phyllaries**	4 to 5 rows, densely pubescent (outer exposed parts)	ca. 5 rows, densely pubescent	4 to 6 rows, sparsely pubescent
**Outer phyllaries**	ovate-elliptic, 7–10 × 3.0–3.5 mm	ovate-elliptic, 16–18 × 1.5 mm	narrowly triangular or ovate-triangular, 7–12 × 2–3 mm
**Inner phyllaries**	narrowly elliptic to linear, 13–15 × 2 mm	broadly linear, 20–22 × 2.3–2.5 mm	linear-narrowly lanceolate, 11–13 × 1–2 mm
**Receptacle bristles**	ca. 4 mm long	6–8 mm long	5–7 mm long
**Corolla (tube / limb with lobes) size**	1.0–1.25 cm (5–7 mm / 4–6 mm with 1.5–2.2 mm lobes)	1.2–2.5 cm (10–13 mm / 6–9 mm with 4–5 mm lobes)	1.2–1.6 cm (4–8 mm / 6–8 mm with ca. 3 mm long lobes)
**Anther**	ca. 5 mm with 1.2 mm tail	ca. 8 mm with ca. 1.5 mm tail	ca. 6.5 mm with ca. 1.8 mm tail
**Achene size**	3.5–4.5 × 1.5–2.0 mm	5–6 mm	4–5 mm

##### Molecular affinities.

**Figure 4.**
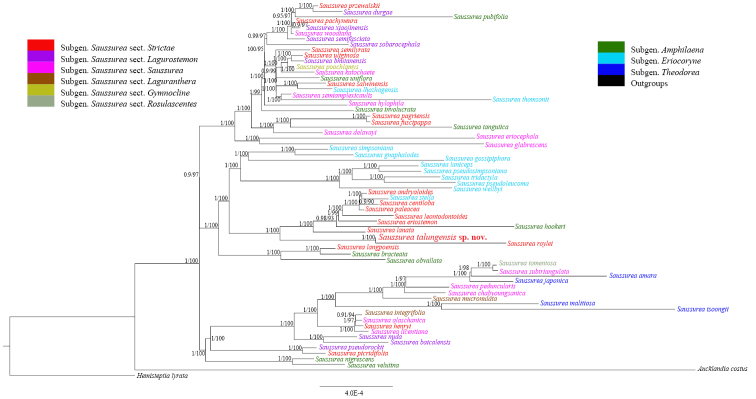
Complete chloroplast genome sequence-based phylogenomic tree inferred from Maximum Likelihood (ML) analyses. Numbers above branches are Bayesian posterior probability (PP)/Likelihood bootstrap support (BS) from BI and ML, respectively.

The typical quadripartite structure of the newly sequenced plastome has size of 152,355 bp (37.7% GC content) consisting of a large single copy (LSC: 83,371 bp, 35.8% GC content), a small single copy (SSC: 18,562 bp, 31.4% GC content), inverted repeats (IRs: 25,211 bp, 43.1% GC contents each of IRA and IRB) (Fig. 1E). The newly sequenced chloroplast genome was used to determine the phylogenomic relationship of *S. talungensis* with its allied species and infer its position within *Saussurea*. The molecular phylogeny through BI and ML tree revealed that *S. talungensis* is nested within a clade comprising *S. roylei*, *S. lanata*, *S. hookeri*, *S. eriostemon* Wall. ex C.B.Clarke, *S. leontodontoides* (DC.) Sch.Bip., *S. paleacea* Y.L.Chen & S.Y.Liang, *S. centiloba* Hand.-Mazz., *S. stella* Maxim. and *S. andryaloides* (DC.) Sch.Bip. (PP > 0.98, BS > 93%; Fig. 4). It is more evident that *S. talungensis* is a sister to *S. roylei* (an allied species), and is supported by Bayesian posterior probability (PP = 1) and Likelihood bootstrap support (BS = 98%) (Fig. 4). Also, complete chloroplast genome structure is conservative in overall size and the order and size of each gene and intergenic region (Fig. 1E). The identical BI and ML phylogenomic tree using plastome sequence revealed that *S. talungensis* is most closely related to *S. roylei* (Fig. 4), which is in congruence with the morphological observations.

##### Additional herbarium specimens examined.

***Saussurea lanata*. Nepal. Jumla**: 3050 m a.s.l., 1952, *O. Polunin et al. 3101* (BM, E); **Mustang**: Tukucha, 3050 m a.s.l., 1954, *J.D.A. Stainton et al. 7846* (BM). **China. Xizang**: Lhozhag, 4450 m a.s.l., 2013, *FLPH Tibet Exped. 13-1617* (PE); Gyaca, 4800 m a.s.l., 1972, *Tibet Chinese Herbal Medicine Census Team 4538* (PE); Gongbo’gyamda, Bahe, 98°59'41.15"N, 93°41'38.35"E, 3350 m a.s.l., 2012, *FLPH Tibet Exped. 12-2144* (PE); Lhunze, Sanga Choling, 28°35'22.92"N, 92°55'12.54"E, 3960 m a.s.l., 2013, *FLPH Tibet Exped. 13-0860* (PE).

***Saussurea roylei*. Pakistan. Gilgit**: 3350–3660 m a.s.l., 1955, *Webster & Nasir 6518* (GH). **India. Himachal Pradesh**: Chamba, Saach Pass, 3960 m a.s.l., 1870, *G. Watt 2158* (K); Kullu, Rotang Pass, 3960 m a.s.l., 1916, *R.E. Cooper 5547* (E); **Uttarakhand**: Tehri Garhwal, Rhuduphera, 3350–3660 m a.s.l., 1883, *J.F. Duthie 857* (K). **Nepal. Dolpa**: Balangra pass, 1952, *O. Polunin et al. 2518* (BM, E); **Bajhang**: Saipal, 4880 m a.s.l., 1954, *J.M.E. Arnold 102* (BM); Guruchi lekh, 3350 m a.s.l., 1990, *N.K. Bhattarai 90/1240* (KATH); **Jumla**: Bhurchula lekh, 3350–3500 m a.s.l., 1952. *O. Polunin et al. 4604* (BM, E); **Manang**: Marsyangdi valley, 4115 m a.s.l., 1950, *D.G. Lowndes 1179* (E, KATH); **Mustang**: Samargaon, 4572 m a.s.l., 1954, *J.D.A. Stainton et al. 7297* (E, KATH); Tukucha, 3050 m a.s.l., 1954, *J.D.A. Stainton et al. 7946* (BM, E, KATH).

### Key to the three closely related *Saussurea* species

**Table:** 

1	Leaf blades oblong to narrowly elliptic, coriaceous, base decurrent; phyllaries 4 to 6 rows, sparsely tomentose, outer phyllaries narrowly triangular or ovate-triangular	***S. lanata***
–	Leaf blades lanceolate, chartaceous, base attenuate; phyllaries in 4(5) rows, densely tomentose, outer phyllaries ovate-elliptic	**2**
2	Stem diameter < 1 cm; leaf margin green; capitula usually 1 rarely 2; receptacle bristles 6–8 mm long; corolla tube 10–13 mm and limb 6–9 mm including 4–5 mm lobes; anthers ~8 mm with 1.5 mm tails	***S. roylei***
–	Stem diameter > 1.3 cm; leaf margin purple; capitula 1 or 3; receptacle bristles ~4 mm long; corolla tube 5–7 mm and limb 4–6 mm including 1.5–2.2 mm lobes; anthers ~5 mm with 1.2 mm tails	***S. talungensis***

## Supplementary Material

XML Treatment for Saussurea talungensis
